# Exploring Vaping Cessation App Use Among Youth: Qualitative Descriptive Study

**DOI:** 10.2196/85778

**Published:** 2026-06-05

**Authors:** Karlee Fonteyne, Mischa Taylor, Roula Nawara, Danielle Rodberg, Tina Afshar, Laura Struik

**Affiliations:** 1 School of Nursing, Faculty of Health and Social Development, University of British Columbia, 1147 Research Road, Kelowna, BC, V1V 1V7, Canada, 1 250-807-9972; 2 School of Population and Public Health, Faculty of Medicine, University of British Columbia, Vancouver, BC, Canada

**Keywords:** youth vaping, vaping, mobile health, digital interventions, mobile apps, qualitative description, public health

## Abstract

**Background:**

Youth vaping has become a significant public health concern, with high rates of initiation, nicotine dependence, and a tendency to underestimate vaping-related harms. Although many youth attempt to quit, most do so without formal support, and few report leveraging cessation apps. Mobile health tools have potential as accessible youth-oriented supports; yet, little is known about youth experiences with these interventions.

**Objective:**

This study aims to explore Canadian youth perceptions and experiences of vaping cessation mobile apps, focusing on preferences, perceived utility, and recommendations for improvement.

**Methods:**

A qualitative descriptive design guided by a constructivist paradigm was used. Overall, 17 semistructured interviews were conducted with youth aged 16‐24 years from British Columbia, Ontario, and Quebec in fall 2022. Eligible participants had current or past vaping experience, had attempted or wanted to quit, and had used a cessation app. Data were transcribed verbatim and analyzed inductively using reflexive thematic analysis, with reflexivity supported through team-based coding and iterative discussions.

**Results:**

Three themes described youth experiences. “Quitting on my Own Terms” reflected the importance of individual agency and commitment to quitting. While there were varied views on apps supporting their agency with cessation, the youth agreed that intrinsic motivation was the most critical element in their cessation journey. “Parallel Experiences- Differing Views on Community” captured ambivalence toward in-app social features: while some valued peer support, others preferred to quit privately or used broader online platforms, like Reddit. “Pocket-sized Partners in Cessation” highlighted the usefulness of quick access to features, such as progress tracking, motivational reinforcement, and gamification, as they engaged in the various stages of quitting (from contemplation through to commitment). However, participants stressed that the convenience of an app was not enough. Youth emphasized the need for apps that feel authentic, emotionally resonate, and are capable of balancing independence with opportunities for connection to social support.

**Conclusions:**

Youth want cessation apps that function as companions to help them leverage their motivation and efforts to quit, rather than as passive trackers. Effective app-based interventions should validate autonomy, account for nonlinear cessation journeys and relapses, and integrate supportive, interactive, and youth-friendly features. By addressing the preferences of youth in the design of vaping cessation apps, digital cessation tools can move beyond convenience to become meaningful companions in youths’ efforts to quit vaping.

## Introduction

### Background

The dramatic surge in e-cigarette, or vaping, use among young people has created a pressing public health crisis across North America, demanding urgent attention and innovative intervention. Data from the United States indicate that in 2023, 10.3% of young adults aged 18‐20 years and 15.5% aged 20‐24 years disclosed vaping regularly [1], while a more recent statistic from the 2024 National Youth Tobacco Survey highlighted that 7.8% of high school students (grades 9‐12) reported vaping in the past 30 days [2]. These youth are drawn to vaping for a variety of reasons, including stress relief, enjoyment, curiosity, social influences, and the perception of vaping as a safer alternative to smoking [3-5].

In both the United States and Canada, vaping products (including those with and without nicotine) are primarily sold “over the counter” at retail outlets such as convenience stores, gas stations, and specialty vape shops. Unlike certain international jurisdictions, North American regulations do not require a doctor’s prescription or pharmacy-based dispensing [6,7]. Instead, access is governed by minimum age laws, 21 years at the federal level in the United States [6], and 18 or 19 years in most Canadian provinces [7], which are enforced through mandatory point-of-sale photo identification checks.

Despite growing evidence of vaping-related health risks, such as respiratory issues, nicotine addiction, exposure to harmful chemicals, and a correlation with negative mental health effects, many youth continue to underestimate vaping-related harms [8-10]. This misperception, combined with the highly addictive nature of nicotine and the powerful influence of peer norms, contributes to patterns of sustained use and dependence. Although many youth express interest in quitting, they often encounter significant obstacles, including limited access to tailored cessation resources, a lack of formal guidance, and uncertainty about where to begin. As a result, supporting youth in their efforts to quit vaping requires interventions that are not only evidence-based, but also accessible, engaging, and responsive to the unique challenges they face [3,5,11-13].

Many youth looking to quit vaping nicotine do so through unassisted strategies such as quitting “cold turkey” or gradually reducing nicotine levels, rather than seeking cessation support or leveraging available resources like quit lines, mobile apps, or SMS text messaging programs [13-15]. Recognizing the urgent need for youth-centered cessation support, public health agencies, researchers, and technology developers have increasingly turned to apps as scalable tools for vaping cessation [16,17]. With 96% of Canadian youth aged 15‐24 years reporting smartphone use and nearly half checking their phones every 15 minutes, mobile health interventions are well-positioned to deliver accessible, real-time, and personalized support [18]. Although youth demonstrate interest in health-related apps, actual engagement with vaping cessation apps remains low. For instance, only 5.9% of current youth vapers who attempted to quit in the past year reported using an app or SMS text messaging program, while 63.7% attempted to quit unassisted [19].

A recent meta-analysis found that digital interventions modestly increased the odds of short-term abstinence but stressed the need for more rigorous, youth-specific research in this space [20]. Further, a review by McKay et al [21] concluded that most vaping cessation apps offer only moderate functionality and often lack critical behavior change elements, such as goal-setting and personalized feedback, which are particularly important for youth engagement. Sanchez et al [22] found that most apps were repurposed from smoking cessation programs and failed to reflect the distinct needs, motivations, and usage patterns of youth who vape. In addition, interactive and gamification-based features, such as progress tracking, rewards, and milestone-based feedback, have been identified as promising strategies to enhance engagement and motivation among youth. Yet, these elements remain inconsistently implemented and rarely evaluated within vaping cessation apps [23-25]. Compounding these design limitations, most vaping cessation apps do not report user metrics, such as downloads or demographic reach, making it difficult to assess their real-world impact. In contrast, smoking cessation apps frequently demonstrate broad uptake, with hundreds of thousands of downloads and targeted promotional campaigns [26]. This lack of comparable data underscores a significant gap: while mobile apps offer high potential for engaging youth in vaping cessation, there is a limited understanding of whether these apps are being used, how they are perceived, and whether they meet the needs of their intended audience [27]. User-centered research is essential to fill this gap and inform the development of more effective, accessible, and relevant digital cessation tools for youth.

### Study Aim and Significance

This qualitative study aims to explore youth users’ perceptions and experiences with using vaping cessation mobile apps. Specifically, we explored how youth engaged with the app, their perceptions of app use, and areas for improvement. These insights are crucial for informing the development of more effective, relevant, and engaging vaping cessation interventions that align with youth’s behavioral, motivational, and technological needs. By centering youth voices, this study contributes to a growing body of research focused on tailoring digital health interventions to vulnerable populations. The findings have significant implications for public health efforts to reduce vaping among youth and young adults, ultimately supporting the promotion of healthier behaviors and long-term well-being.

## Methods

### Study Design

Drawing on its alignment with a constructivist worldview where subjective realities exist and can be known through subjective accounts of individual experiences [28], we used qualitative description to explore this topic. Qualitative description is a methodology useful for exploring and describing phenomena that remain understudied, while staying close to the data in its interpretation [29].

### Sampling and Recruitment

A purposive sampling strategy was used to recruit participants whose lived experiences could meaningfully inform the research questions. Eligible participants were youth aged 16 to 24 who met the following criteria: (1) currently vape or have vaped in the past, (2) are currently or were previously motivated to quit, (3) have used or are using a vaping cessation app, (4) reside in Canada, and (5) speak English fluently.

Recruitment was conducted via social media platforms, specifically Instagram (Meta) and Facebook (Meta), where a digital advertisement invited youth to share their experiences with quitting or attempting to quit vaping in a study led by researchers at the University of British Columbia (UBC). The ad included a QR code and a hyperlink to a brief eligibility survey with 5 “yes” or “no” questions. The research team reviewed responses and compiled a list of eligible participants, none of whom were known to the team prior to the study.

Efforts were made to ensure gender balance through a recruitment ad that used gender-neutral language and imagery. During recruitment, we monitored the gender representation of the incoming sample to assess whether we were meeting our gender-balancing target. Ultimately, the sample consisted of 10 self-identified women and 7 self-identified men. Eligible participants were contacted via email with an invitation to participate, a demographic questionnaire, and a request for their availability. Interviews were scheduled after written consent was obtained. At the start of each interview, informed and ongoing consent was reviewed with participants.

### Data Collection

The interview guide was developed collaboratively by the research team in consultation with experts in the field and a youth co-researcher (Multimedia Appendix 1). It was circulated among team members for review and revisions before finalization. Interview questions for this study explored participants’ experiences of the app in relation to their quitting goals, such as “What prompted you to download a vaping app?” “What features on the app kept you coming back?” and “Which social support features did the app offer, and what was your experience of using them?” This study draws on data collected from September to October 2022.

Overall, 17 semistructured interviews were conducted online via UBC-licensed Zoom in September and October 2022, with participant consent. The research team was based in British Columbia, Canada, while participants were located across British Columbia, Ontario, and Quebec. Interviews were conducted by 1 of 4 trained female research assistants and lasted 30‐60 minutes. Interviewers adhered to the semistructured guide while allowing participants to elaborate on their experiences. Recruitment and interviewing occurred concurrently, with recruitment concluding once the team determined that data saturation had been reached and no new insights relevant to the research question were emerging.

### Data Analysis

Following the completion of all interviews, deidentified audio recordings were uploaded to Otter.ai for transcription. Each transcript was reviewed by 2 research assistants (RN and DR) to verify transcription accuracy and to correct any discrepancies. To facilitate initial familiarization with the data, RN and DR organized the interview content by mapping responses to their corresponding interview questions using NVivo 14 (Lumivero). The document was subsequently circulated among the research team to provide an initial overview of the content derived from the interviews.

An inductive thematic approach was used to interpret the data. Following an iterative, collaborative process, the research coordinator (MT) and research assistants (RN and KF) engaged in reflexive thematic analysis as outlined by Braun and Clarke [30-32]. This approach adhered to the 6 foundational phases of thematic analysis originally described by Braun and Clarke [32]: familiarization with the data, generating initial codes, searching for themes, reviewing themes, defining and naming themes, and producing the final report. However, consistent with reflexive thematic analysis principles, the analysis was conducted with greater flexibility and interpretive depth. Rather than treating themes as objectively “found” within the data, the team actively constructed themes through an ongoing, reflexive engagement with the material [30-32].

To support a meaningful exploration of participants’ experiences beyond the constraints of the interview questions and allow for true inductive analysis, the research team conducted initial coding directly from the original participant transcripts rather than the NVivo document that organized data by interview question. This step was conducted using Microsoft Excel due to compatibility issues between NVivo 14 and the diverse operating systems (PC and macOS) used by team members. MT and RN developed an initial coding framework by inductively analyzing the first 4 transcripts, which was then shared with other team members for feedback. Following an iterative process, as new codes emerged during analysis of the remaining transcripts, the coding framework was updated and circulated among the team for discussion. Initial codes alongside the corresponding extracted participant quotes were compiled in a master Excel document. Research team members independently reviewed the master Excel document to identify emerging patterns and conceptual throughlines that could form overarching themes. KF and RN then collaboratively synthesized these individual analyses, identifying cross-cutting patterns and refining them into consolidated themes. Members of the research team regularly met to review and refine these themes into findings. To ensure rigor and trustworthiness, the team maintained detailed audit trails, engaged in regular analytic discussions to question interpretations and surface assumptions, and practiced continuous reflexivity throughout the coding and theme development process [32]. This reflexive approach enabled a richer and more nuanced interpretation of participants’ experiences.

### Ethical Considerations

This study was granted ethics approval by the UBC Okanagan Behavioral Research Ethics Board (#H22-01408) in the summer of 2022. The recruitment process and all interview sessions occurred concurrently in the fall of 2022. Informed consent was obtained from all participants, which was voluntary and revisited throughout the research process. Participants were provided with information about the study and a digital consent form emphasizing voluntary participation, the study’s purpose, potential risks, and the right to withdraw at any time without consequences. Given that the legal age to provide informed consent is 16 in Canada, parental consent was not required for this sample. Youth were provided with an email address to contact a researcher with additional questions or to request more information about the study. Each participant received a CAD $50 (US $36) e-gift card for their time and contributions.

The study implemented a comprehensive data management plan to ensure the secure handling, storage, and organization of research data. All data collected, including audio recordings, transcripts, and questionnaires, were stored on password-protected UBC servers in Canada and accessible only to the research team. During data collection, audio recordings from interviews were securely uploaded to a cloud-based storage system that complied with UBC’s institutional data security policies. These recordings were transcribed verbatim, with all personally identifiable information removed to maintain participant confidentiality. Participants were assigned a participant ID# (including gender and age), and only this anonymized identifier was included in the transcription, data files, and interview notes. Transcripts and associated metadata were organized and stored in NVivo 14, a qualitative data analysis software platform that provides secure data coding and retrieval tools. After the study concludes, data will be retained for 5 years in accordance with ethical guidelines, after which all files will be securely deleted. Participants were informed of these measures during the consent process to ensure transparency.

## Results

### Participant Characteristics

The final sample comprised 17 participants, the majority of whom identified as women (10/17, 58.8%), with the remainder identifying as men (7/17, 41.2%; Table 1). Most participants were aged 18‐24 years (14/17, 82.4%), with a smaller proportion aged 16‐17 years (3/17, 17.6%). Participants were primarily from British Columbia (7/17, 41.2%) and Ontario (6/17, 35.3%), with fewer from Quebec (4/17, 23.5%). Vaping history varied, though the largest proportion reported vaping for 4‐5 years (7/17, 41.2%), followed by less than 2 years (6/17, 35.3%). Most participants reported vaping several times per day (11/17, 64.7%), and a substantial majority indicated they were currently trying to quit (13/17, 76.5%). Nearly half (8/17, 47%) had attempted to quit 2‐5 times, while 35.3% (6/17) reported a single quit attempt. Social exposure to vaping was high, with most participants indicating that some (7/17, 41.2%) or most (8/17, 47%) of their friends vape.

**Table 1.** Demographic table.

Demographic and Categories	Values, n (%)
Sex (Gender)	
Female (woman)	10 (58.8)
Male (man)	7 (41.2)
Age range (years)	
16‐17	3 (17.6)
18‐24	14 (82.4)
Ethnicity	
Asian	4 (23.5)
Non-Hispanic White	6 (35.3)
Black	4 (23.5)
Latino	1 (5.9)
First Nation	1 (5.9)
White and Asian	1 (5.9)
Province of residence	
British Columbia	7 (41.2)
Ontario	6 (35.3)
Quebec	4 (23.5)
Highest education	
Less than high school	2 (11.8)
High school diploma	8 (47)
College or trades	6 (35.3)
University degree	1 (5.9)
Length of time vaping nicotine in years	
<2	6 (35.3)
2‐3	2 (11.8)
4‐5	7 (41.2)
≥6	2 (11.8)
Currently trying to quit	
Yes	13 (76.5)
No	4 (23.5)
Frequency vaping	
Several times/day	11 (64.7)
Once/day	3 (17.6)
A few times/week	3 (17.6)
≤ Once per week	0 (0)
Quit attempts	
Once	6 (35.3)
2‐5 times	8 (47)
6‐10 times	2 (11.8)
>10 times	1 (5.9)
Number of friends who vape	
None of them	0 (0)
Some of them	7 (41.2)
Most of them	8 (47)
All of them	2 (11.8)

Findings revealed that youth experiences with vaping cessation apps were shaped by individual motivations, preferences, and perceptions of support. Three overarching themes emerged. “Quitting on My Own Terms*”* reflected the importance of autonomy and self-motivation, with youth expressing varied views on whether apps enhanced or undermined personal responsibility. “Parallel Experiences- Differing Views on Community” captured divergent perspectives on social support features; while some saw value in connecting with others, many preferred to quit independently or questioned the usefulness of app-based communities. Finally, “Pocket-Sized Partners in Cessation” highlighted the practical and motivational support apps provided across the quitting journey, though the subtheme “Caveat: Convenience Alone Is Not Enough” emphasized that basic features and design choices often limited their impact. These themes reflect how youth users perceive vaping cessation mobile apps, as well as their experiences with using them. These themes were shared across the sample, with no gender nuances detected between women and men.

### Theme 1: Quitting on My Own Terms

#### The Tension Between Individual Responsibility and Leveraging App-Based Support

Participants commonly described quitting vaping as an intensely individualized process; yet, there was notable variability in how they interpreted what it meant to “quit solo.” For some, using an app did not conflict with their desire to quit independently. These participants viewed the app as a nonintrusive aid and as a resource that helped them maintain accountability and structure without undermining their personal agency. As one participant explained:

I’ve tried everything that I can do myself separately. I don’t try to rely on external help [...] it’s an app helping it’s not anyone in person.

[Participant #10, Male]

Another participant valued app-based support for being nonjudgmental:

[it’s a] guilt free approach,” appreciating “the fact that it doesn’t judge.

[Participant #19, Male]

For some, the app’s neutrality and lack of judgment made it a uniquely safe space to seek support without compromising their sense of independence. In these cases, apps served as a bridge between unsupported quitting and fully assisted interventions, preserving the autonomy that many youth valued while still offering practical guidance.

Conversely, other participants saw any reliance on external tools, including apps, as diminishing the self-responsibility they felt was essential to quitting. For these individuals, using an app was seen as inconsistent with the values of independence and self-control. One participant conveyed this sentiment clearly:

I feel like people need to have more self-responsibility, especially if you’re an adult.

[Participant #7, Female]

However, this participant acknowledged that apps may be more suitable for other age groups, continuing, “[apps may be] really good for teens.” Participants described their desire to quit independently, with some preferring greater or lesser degrees of app-based support.

#### Motivation as the Fuel: The Role of Personal Commitment

Across interviews, a key message emerged: self-motivation was the fuel in driving the cessation process. Participants frequently described how vaping cessation apps required a high degree of intrinsic motivation, and that their effectiveness was directly tied to the user’s personal readiness and commitment to quit. While this was viewed positively by those who appreciated that the app reinforced their self-driven efforts, others saw it as a potential shortcoming. For youth who were not fully committed, the app alone was unlikely to spark or sustain change. As one participant put it:

If anyone’s like, willing to quit, like an app is definitely […] your go to, in the beginning, I feel like it could be really helpful and like, self-encouragement, because like, although like the app is there to help you, you kind of have to be self-motivated.

[Participant #9, Female]

According to this participant, apps can provide helpful structure early on but must align with an existing desire to quit to be effective.

Apps were often described as tools that facilitate but cannot initiate change. They offered encouragement, goal tracking, and health tips, but these features only resonated when a user was genuinely ready to take action. Many participants emphasized that true success in quitting required deep internal resolve, regardless of external supports. For these youth, a central condition for cessation was personal determination, not the tools used:

I think if you’re really going to quit it as it has to come from, you know, you personally, it’s you, you have to take that decision [..] not to fall back into your addiction.

[Participant #4, Female]

As another participant succinctly explained:

I do think it can help out a lot. But only if you’re serious about it.

([Participant #2, Male]

For these participants, apps were considered a supportive tool to accompany their cessation journey following their personal dedication to quitting.

### Theme 2: Parallel Experiences—Differing Views on Community

Participants expressed divergent attitudes toward engaging with app-based vaping cessation communities, reflecting a range of personal preferences, needs, and beliefs about the role of social support in quitting. Engagement with in-app communities was shaped by personal values, perceived credibility, stigma, and the app’s design choices. While many youth spoke positively about the concept of in-app social features, others conveyed reluctance or disinterest.

For some youth, there were clear benefits of social connection. These participants highlighted how in-app communities could offer emotional validation, encouragement, and a sense of shared experience during vulnerable moments. One participant emphasized how peer connection could serve as a powerful reminder during times of struggle:

You forget that there are people around, you tend to forget that you're not alone. It’s like it can, you just can become trapped in a world of misery. And it’s like a reminder that you're not alone. Sometimes you need to see it.

[Participant #6, Female]

Others valued the opportunity to hear about shared experiences, which helped normalize their own journey and served as a source of encouragement:

When you see someone is actually getting better or doing better, you might be encouraged, you want to try to, since this person is doing it, I too can do it.

[Participant #17, Male]

Another participant emphasized the value of peer connection, especially when dealing with social pressure:

Sometimes it’s hard. Especially with all the social pressures around you, it’s easy to swerve off track. So I think having an app and having, like, people that you can visibly see are going through the same thing as you has been really great.

[Participant #11, Female]

For these participants, the sense of connection and affirmation that accompanied app-based social support features was meaningful and valuable.

At the same time, some participants opted not to engage with in-app social features, either due to a general lack of interest or a preference for managing cessation as a self-directed, private process. For some, connecting with others around quitting vaping felt unnecessary or even uncomfortable. One participant shared:

I would say that I wasn’t even that open to finding this community in the first place. I kind of just, it’s a personal thing, right? […] I’ve always been the type of person where it’s like, if I do something like, I’ll be proud of myself, I don’t really need that community to show off like that I’ve quit nicotine or anything […] It’s embarrassing when you want to quit something you've tried so hard to quit, and you can't like it’s not something really I want to share with the community.

[Participant #8, Male]

Another echoed a similar sentiment when questioned about in-app social support features and highlighted their preference for alternative online discussion platforms where they felt they could engage more organically or anonymously, stating:

I won’t see myself using it. Like, for example, it was like a buddy system […] it could definitely be beneficial to some people, but I feel like for me, I’m just going through it alone. So like, if I want to talk to someone, I could use other apps like Discord or Reddit that can help me you know, connect with some people that they’re going to the same thing as me.

[Participant #2, Male]

Some participants who did not wish to use social support features themselves or had not personally engaged with them often demonstrated an empathetic and theoretical awareness of their potential value to others. These participants recognized that not everyone has access to social encouragement outside of the app and saw how peer connections could serve as a critical resource. As a participant explained:

I wasn’t really into like, I guess connecting with other people, but I can see how it can really help some people if they’re also going through the same thing, and you can connect with them and know that you’re not alone and you have that added support that you may not have otherwise.

[Participant #1, Female]

Beyond personal preference, some participants questioned the effectiveness and credibility of the social features offered by cessation apps. Concerns were raised about community size, visibility, and the perceived lack of investment from app developers in fostering these spaces. One participant explained:

If I wanted to find a community for like anti-vapers, or the non-smokers, it would be easier to go on the internet and like, find that community rather than this, like having to download this random app, and just assume they have a good community on there.

[Participant #8, Male]

They elaborated on how the lack of promotion or visibility signaled a weak or undeveloped social support feature:

I feel like when there is a big community, the company will advertise that a lot. And like, for this app, it just seemed like such a small part of the app. So, through that, I could already tell that, like, there wasn’t really an aspect of the app that like the developers probably worked on a lot or were proud of.

[Participant #8, Male]

For this youth, the value of an app’s social feature was influenced by their perception of its importance to developers. When community tools appeared secondary or underpromoted, some participants dismissed them as inauthentic or untrustworthy. Participants shared a range of experiences and perceptions of app social support features. While some youth found value in shared experiences and forming community with others, others preferred to manage their quit journeys privately or through broader online platforms.

### Theme 3: Pocket-Sized Partners in Cessation

#### Support From Contemplation to Commitment

Vaping cessation apps were viewed as multifaceted tools that offered support across the continuum of quitting, from initial contemplation to sustained commitment. The early phases of quitting were supported through app features that reinforced the decision to quit by highlighting health and financial benefits and prompting users to reflect on their personal motivations. Several youth appreciated the inclusion of positively framed, evidence-based information about how the body recovers after quitting, which helped dispel misconceptions and instilled hope. For these youth, the apps provided affirmation that change was both possible and worthwhile.

As participants progressed in their cessation journeys, visual depictions of progress emerged as particularly valued features. These included stats like time vape-free, money saved, and improvements in health, presented in clear, tangible formats that users found both satisfying and motivating. One participant shared:

[The app] tells me how much money I’ve saved. And it also includes goals, which I really like, because I'm pretty cheap when it comes to stuff. So when it tells me I've saved right now, it says $86. That is enough motivation for me to continue to stop in itself.

[Participant #11, Female]

This positive tone was especially helpful during moments of craving or doubt. For many, being able to see how far they had come helped them resist relapse and regain motivation. Participants also appreciated apps that celebrated small wins, such as weekly milestones or progress trophies. These features helped build momentum and a sense of accomplishment. A participant shared:

Trophies they give you at every week, whether you’re vape free, so it’s, you know, it can it helps you keep motivated to not start again. It’s like a small thing. But you know, for me like, it’s, it’s made a big difference.

[Participant #6, Female]

These rewards, though seemingly minor, were perceived as meaningful validations of effort, reinforcing continued engagement with the app. With that in mind, participants were dismayed by any overly punitive loss of progress. One youth highlighted this saying:

The problem with it, I would say, is that you have like a huge streak and then if you hit it [vape], like once it’s like, you lose all of it.

[Participant #15, Male]

Overall, participants emphasized the importance of positive reinforcement and personalization in maintaining their motivation throughout their quit journeys. Apps that provided motivational content, celebrated progress, and allowed personal goal setting were described as accessible and emotionally resonant, making the quitting process feel more achievable.

#### Caveat: Convenience Alone Is Not Enough

Although participants appreciated the ease and accessibility of app-based cessation tools, many emphasized that convenience alone was insufficient for sustained engagement or meaningful support. They were clear that vaping cessation apps must be thoughtfully designed with youth-specific needs and experiences in mind. Several participants described glitches or shallow content that felt disconnected from their real-life experiences of addiction and quitting. These limitations made the app feel more like a passive tracker than an active support system. Participants frequently critiqued basic or redundant features, such as simple day counters, as unoriginal and easily replicable. A participant explained:

I can always on a piece of paper, I could track on the days that I’m not vaping. Right, so like, even though that part of the app is helpful, like it’s not really anything special. App should incentivize more to keep people accountable.

[Participant #8, Male]

Others suggested that an app was limited in its ability to provide comprehensive cessation support. Apps that functioned solely as stand-alone tools were perceived by some participants as insufficient, particularly in vulnerable moments of their cessation journeys. One participant noted:

The app does not work well on its own; it needs to be accompanied by some form of social support to act as an external motivator. It did help in the beginning, like to be honest, like, but I feel like the app plus something else would be like a better option. I feel like the app itself is not good as a singular method to stop.

[Participant #12, Female]

For these users, superficial tracking tools lacked the motivation or engagement needed to sustain change. To improve effectiveness, participants recommended more dynamic and personalized features, such as daily (rather than monthly) progress updates, visually expressive milestones, and uplifting motivational rewards. Youth wanted apps to feel not only functional but also affirming, something that could reflect and celebrate their efforts in real-time. To this point, one participant voiced their experience, stating:

When I, I feel like, I feel that that that need coming up? that like itch to vape. You know, I opened the app, and I look at those reminders, and it helps me control those urges.

[Participant #4, Female]

Some participants were particularly drawn to gamification as a mechanism to increase engagement. These features reframed cessation as a challenge or interactive experience, transforming the quitting journey into something more fun and rewarding. One participant shared:

Overall, like, I really liked the whole concept of the app, like how it’s kind of like a game, I feel like, you get you like, unlock like, like these milestones for completing like, like cessation-related tasks.

[Participant #9, Female]

Another echoed this enthusiasm:

We’re having fun tasks that look like a game or something [...] the gamification obviously kept me coming back because it was fun.

[Participant #19, Female]

These features helped keep youth invested, particularly during periods when intrinsic motivation may have wavered.

In addition to gamified elements, some participants expressed interest in extrinsic motivators, especially financial incentives. They suggested that tangible rewards could offer meaningful reinforcement, particularly for youth facing limited access to traditional support systems.

It would be cool if there’s like a point system where, like, I don’t know, if you got like 20 cents for like, five months going vape free or something like that […] it would be cool, because help isn't like the only motivation, there are other motivations, like saving money.

[Participant #10, Male]

These suggestions bring forward some material elements that youth want to see in a cessation app to make it worthwhile.

Beyond incentives and features, some youth highlighted the absence of connection to human support, particularly from health professionals. While digital tools were seen as useful, they did not replace the relational aspects of care. One participant expressed a desire for real-time, human interaction, saying:

[I'd] love to have the option of speaking to someone […] Even if we're not going to show faces just someone, not a bot, but an actual human being who I can talk to, even if I have to pay for the services. Because I think what people are trying to avoid is meeting people in person.

[Participant #14, Male]

This participant valued empathy and responsiveness as integral to achieving their cessation goals, elements that they perceived were difficult to replicate through static app content.

Finally, participants emphasized that apps should more authentically mirror the lived realities of youth navigating addiction. As one participant put it:

Your cell phone, as much as you’re on it, that’s not your real life.

[Participant #7, Female]

This youth acknowledged that, as digital tools, cessation apps are inherently distinct and separate from their lived experiences and therefore limited. Youth called for content that acknowledges the emotional, social, and identity-related complexities of quitting, including real-time chat, peer stories, or spaces to share experiences. They expressed a need for tools that go beyond tracking behaviors and instead foster a sense of connection, relatability, and real-world relevance.

## Discussion

### Main Findings

This study explored youth experiences with vaping cessation apps and identified 3 interrelated themes that reflect the complexity of digital cessation support. The first theme, “Quitting On My Own Terms,” reflected the youth’s emphasis on autonomy and personal responsibility in the quitting process. While some youth found that app-based tools aligned with their sense of independence, others viewed any external aid, even a self-guided app, as misaligned with their belief in “quitting solo.” The second theme, “Parallel Experiences- Differing Views on Community,” revealed divergent perspectives on app-based social support features. Some participants valued community engagement and peer stories, whereas others expressed skepticism about their relevance and opted not to engage, preferring independence or alternative platforms. Finally, “Pocket-Sized Partners in Cessation” described youth preferences for apps that offer visual feedback, motivational reinforcement, and personalized encouragement through the different stages of cessation. Yet, participants were clear that convenience alone was insufficient; apps must feel authentic, emotionally resonant, and relevant to the realities of quitting vaping.

Taken together, these findings suggest that youth want tools that affirm their self-awareness, validate their autonomy, and simultaneously offer the option of meaningful connection and support. Apps that appear overly simplistic, technically flawed, or disconnected from youth’s lived realities are unlikely to foster sustained engagement. Instead, youth called for interactive, affirming, and emotionally intelligent tools, pocket-sized partners that do more than count days; they recognize and account for the complexity of change.

These findings align with prior studies demonstrating that youth value flexible, personalized features, behavior tracking, and positive reinforcement in digital health interventions [13,24,25,33-35]. Consistent with Bold et al [33], participants emphasized the importance of supportive rather than condescending messaging. While prior research has highlighted personalization and community support, participants in this study related the central role of autonomy in cessation efforts. For some, even minimally directive features felt misaligned with their belief in quitting independently. While moderated online communities have been previously identified as desirable [12-14,35,36], participants expressed a degree of ambivalence and skepticism, underscoring the need for apps that prioritize social support features to ensure these communities are meaningful and thoughtfully integrated. A novel contribution of this study is the critique that convenience alone is not enough. Youth distinguished between apps that merely track progress and those that offer authentic, emotionally validating support. This insight suggests that digital cessation interventions must extend beyond functionality to resonate meaningfully with youth’s lived experiences.

From a developmental perspective, adolescence and emerging adulthood are marked by identity formation, autonomy, and peer influence [37]. Aligning interventions with these developmental needs, via supporting independence alongside offering pathways for connection, may strengthen effectiveness and engagement. Youth in this study also demonstrated a pragmatic view of health behavior change, acknowledging personal responsibility while recognizing the difficulties of cessation. This realism presents opportunities for interventions that walk alongside youth, rather than offering prescriptive solutions.

### Implications for Intervention Design

These findings offer several implications for the design of youth-centered cessation apps. First, interventions should incorporate features that validate youth self-reflection and agency. Positive reinforcement, affirming language, and recognition of progress may enhance engagement [12,24,25,34]. However, autonomy must be balanced with the normalization of help-seeking. Apps should frame support-seeking not as a weakness but as a strength, reinforcing that quitting independently and seeking help are not mutually exclusive.

As a means toward this end, social features must be intentionally designed within the app to boost credibility and the likelihood of engagement. Evidence indicates that a supportive community is a predictor of successful nicotine cessation [36,38,39]. Participants in this study expressed a desire to connect with others who could relate to their personal experiences of cessation and gather encouragement from a supportive community. Yet, participants also expressed skepticism about the quality of the app’s social support features and, subsequently, about the robustness of the community and the support offered. Some participants expressed greater interest in joining cessation communities on larger, more established platforms such as Reddit and Discord. A study of a Facebook cessation support group found that strong leadership by moderators, which stimulated discussion, increased user engagement [40]. Consequently, there is an opportunity for apps to improve their value and effectiveness by investing in and prioritizing social support features, so that they adequately meet the expressed desire of youth to connect with others who are also attempting to quit. If cessation apps were to likewise use moderators or mentors to guide and support discussions on their social features, this could be one step toward increasing engagement and boosting credibility, with the potential to initiate a snowball effect of engagement. Given the connection between a supportive community and cessation success, the social support features of apps should not be an afterthought, but an intentionally designed and marketed central feature.

Third, relapses and setbacks need to be anticipated and normalized as part of the quitting journey. Youth in this study did not commonly account for relapse, and most apps lacked relapse-responsive features to address nonlinear cessation trajectories. This gap is concerning, as lapses are common among youth who attempt to quit [14] and, if not supported, may lead to disengagement. Relapse-responsive features such as customizable recovery plans, self-reflective journaling, adaptive goal setting, and encouraging messaging during setbacks may reframe lapses as learning opportunities rather than failures. Incorporating such features may also strengthen self-efficacy, a key component of cessation as voiced by participants, by reminding users that quitting is a process rather than a one-time decision.

Fourth, hybrid models that combine digital autonomy with access to real people may be particularly valuable [34]. While many youth want to quit independently, others indicated that relational depth and accountability could enhance motivation. Linking users to counselors, quit coaches, peer mentors, or live chat support could provide individualized guidance while preserving choice and autonomy. In parallel, design elements such as gamification, milestone-based incentivization, and progress tracking may sustain attention and reinforce positive behavior change over time [24,25]. Importantly, these features must move beyond superficial rewards to genuinely celebrate progress and reinforce resilience, thereby creating a sense of partnership between the user and the app.

### Strengths and Limitations

This study provides a nuanced understanding of how youth experience and interpret vaping cessation apps, offering important insights for future digital health intervention design. The sample included representation from both young women and men, as well as geographic diversity across Eastern and Western Canadian provinces. These characteristics enhance the breadth of perspectives captured and contribute to the contextual richness of the analysis. The study also benefited from a rigorous qualitative approach grounded in qualitative description [29], which allowed for attention to individual variation while identifying common patterns across experiences.

The researcher’s subjectivity was viewed as an analytic resource rather than a bias to be minimized, and movement between phases was fluid rather than strictly linear. Reflexivity was further supported through consideration of the researcher’s positionality. The research team consisted of qualitative public health researchers with varying levels of experience in tobacco and vaping-related research. The principal investigator (LS) is a senior scholar with extensive expertise in tobacco, nicotine, and vaping research. Team member (KF) is an emerging scholar in nicotine cessation, while MT, RN, and DR are trainees in vaping research. Several of the research assistants were close in age to participants, which may have facilitated rapport-building during interviews and promoted more candid sharing. These intersecting identities and disciplinary backgrounds shaped the analytic lens through which data were interpreted.

Some limitations must be acknowledged. The sample did not include noncisgender individuals, and perspectives from youth living in several Canadian provinces remain unrepresented. As a result, the findings may not fully reflect the experiences of gender-diverse youth or those living in other regional or cultural contexts, thereby limiting transferability. In addition, because the study was embedded within a larger project focused specifically on app users, the findings may reflect the unique experiences and motivations of youth who are already open to using digital tools for cessation. Furthermore, data on the use of other nicotine products (eg, smoking, nicotine pouches, or nicotine replacement therapy) were not collected from this sample. Even further, the data reflect the perspectives of youth in 2022. Given the rapid evolution of vaping products, mobile health technologies, and regulatory environments, future studies will be crucial in exploring how youth perspectives shift over time.

### Conclusions

Youth desire cessation apps that are authentic, emotionally intelligent, and responsive to the complexities of quitting vaping. Effective interventions must validate autonomy while encouraging safe connections, normalize setbacks as part of the quitting process, and integrate features that feel credible, supportive, and youth-friendly. By addressing the preferences of youth in the design of vaping cessation apps, digital cessation tools can move beyond convenience to become meaningful companions in youths’ efforts to quit vaping.

## Supplementary material

10.2196/85778 Multimedia Appendix 1 Semistructured interview guide.

## Abbreviations

UBC: University of British Columbia
