# Supplementary material for: Exploring Vaping Cessation App Use Among Youth: Qualitative Descriptive Study
Source: J Med Internet Res. 2026 Jun 5;28:e85778. doi: 10.2196/85778 (PMC13240636; doi:10.2196/85778)
Supplement: Multimedia Appendix 1 [file jmir-v28-e85778-s001.docx]

**Multimedia Appendix 1**


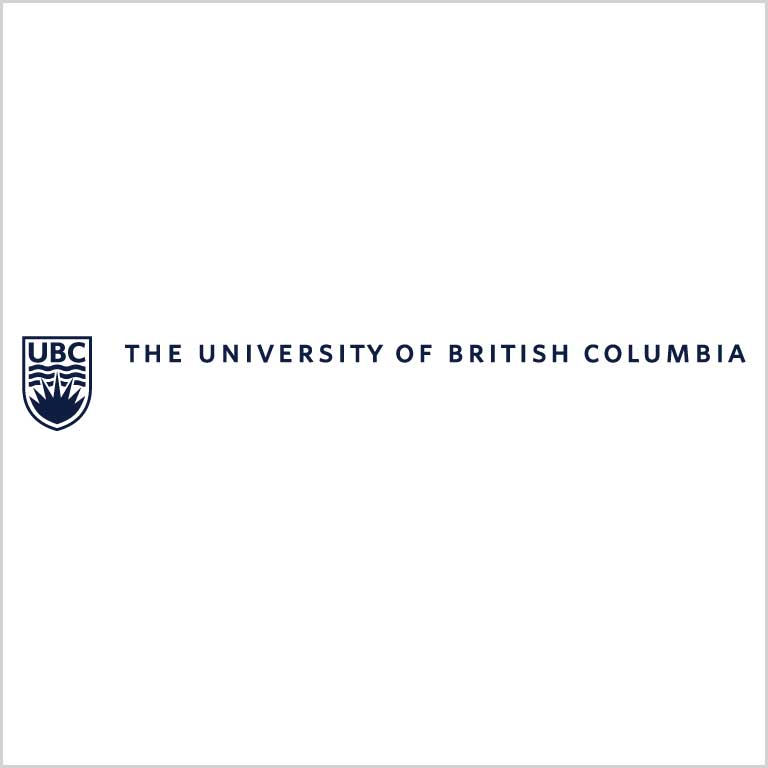
**INTERVIEW GUIDE**

**Project Title: Socio-environmental Influences on Quitting E-cigs (SIQuE) Project**

**Purpose:** Invite young Canadians to share their experiences using a vaping cessation app and discuss their perspectives on how their social context has influenced their vaping cessation. (~45-60 minutes).

| **Preparation (using Zoom for interviews)** |
| --- |

1. AV check
2. Recording ready
3. Materials for sharing uploaded
4. Facilitator notes
5. Ensure consent has been received from participant
6. Ensure demographics survey has been filled out or fill out with participant prior to interview

| **Welcome/Instructions for Facilitators (5 minutes)** |
| --- |

Participants will be welcomed to the interview. Before the interview, we will remind participants that sessions are recorded and will verify that we have their permission to record. They will be reminded of the purpose of the study and that participation is voluntary. Participants will be asked if they have any questions before the recording/interview questions begin.

***Check your email and OneDrive to see if the pre-interview questionnaire has been completed. If it has not been completed, please screen share the document (available in the OneDrive folder) and assist the participant with filling this out prior to the interview.

| **Interview questions (~35 minutes)** |
| --- |

*I have some questions about your experience using a vaping cessation app.*

| **Interview Focus** | **Questions:** |
| --- | --- |
| ***Rapport/contextualization*** | 1. How many apps have you tried for quitting vaping and what were the apps called? (Information available on questionnaire form, can use this question to clarify) 2. Do you mind telling me a bit about your experience with the app you used? For example, how you learned about it, if you’re still using it or not anymore. |
| ***Reasons for Downloading*** | 1. What prompted you to download a vaping cessation app? 2. Is there a reason you chose your specific app compared to other available vaping or smoking cessation apps? |
| ***Usage of App*** | 1. During the time period that you used the app, how often would you open the app to use its services? |
| ***Likes and Dislikes*** | 1. What features on the app kept you coming back? What was it about them that kept you coming back? 2. What features on the app did you dislike? What would you change or what would you add? |
| ***Social Support Components*** | 1. Did the app offer any forms of social support, like chat features or forums? 2. To follow up from the previous question: If yes, which features and what was your experience using them? If no, did you find you missed that aspect of social support and what social support features would you have liked to see? |
| ***Overall Thoughts and Recommendations*** | 1. Overall, how would you describe your experience using a vaping cessation app? Did you find it useful and if you had to go through quitting again would you still use an app? 2. Would you recommend using a vaping cessation app to others interested in quitting vaping? 3. Is there anything else you would like to add in relation to your thoughts on using a vaping cessation app? 4. Are there any additional features (social or otherwise) that you would like within or external to the app to support you quitting vaping? |

*Note that the interview continued with questions about the youth’s social environment and those involved in their lives while they were trying to quit. These guiding questions have been omitted from this document as the collected data were not pertinent to this publication.*

| **Closing: final comments (5 minutes)** |
| --- |

Thank you for joining me today and helping us understand more about the experiences of young Canadians using vaping cessation apps and what social influences in your life are impacting your experience quitting. This information will help in the development of helpful cessation apps and help produce resources that support youth and young adults like you with quitting vaping.

Would you be interested in being contacted to participate in any future studies? Would you like to receive the results of the study? What e-gift card would you like to receive? The options are Amazon, Starbucks, Tim Hortons, PC Optimum, Walmart, Lululemon and EB Games.

We are at the end of this session but before we leave, do you have any final thoughts that you’d like to share?

THANK YOU
